# The impact of disclosing emotions on ratings of interpersonal closeness, warmth, competence, and leadership ability

**DOI:** 10.3389/fpsyg.2022.989826

**Published:** 2022-12-13

**Authors:** Vera U. Ludwig, Blaire Berry, Jerry Y. Cai, Nai Ming Chen, Damien L. Crone, Michael L. Platt

**Affiliations:** ^1^Department of Neuroscience, Perelman School of Medicine, University of Pennsylvania, Philadelphia, PA, United States; ^2^Wharton Neuroscience Initiative, the Wharton School, University of Pennsylvania, Philadelphia, PA, United States; ^3^Department of Marketing, McCombs School of Business, University of Texas, Austin, TX, United States; ^4^Department of Psychology, Northeastern University, Boston, MA, United States; ^5^Marketing Department, the Wharton School, University of Pennsylvania, Philadelphia, PA, United States; ^6^Department of Psychology, School of Arts and Sciences, University of Pennsylvania, Philadelphia, PA, United States

**Keywords:** emotion, self-disclosure, vulnerability, connection, closeness, warmth, leadership, relationships

## Abstract

**Introduction:**

Increasingly, business leaders and other professionals are called upon to be vulnerable and authentic in the workplace, which often includes disclosing emotions to others. While sharing emotions is known to enhance closeness, several questions remain underexplored. Specifically, disclosing personal facts about oneself and disclosing emotions have often been studied together, making it difficult to determine the effects of disclosing emotions *per se*. Moreover, not enough is known about factors that may influence effects of disclosing emotions, including recipients’ attitudes toward emotion-sharing, the sharer’s gender, and whether one considers the disclosure to be similar to one’s own experiences. We examined the impact of disclosing positive and negative emotion on ratings of closeness, warmth, competence, and leadership ability.

**Methods:**

119 participants (95 female) in the United States were shown headshots of individuals who were introduced in the first person in written format. For half of the pictures, an autobiographical fact about the individual’s past was disclosed. For the other half, an autobiographical fact and an associated emotion were disclosed.

**Results:**

We found that sharing both positive and negative emotions increased feelings of closeness above and beyond the effects of autobiographical sharing alone. Sharing positive emotions also increased ratings of warmth, competence, and leadership ability. Male and female sharers benefited equally from disclosing emotions and effects were largely robust to recipients’ attitudes toward emotional expression. Having something in common with the disclosed fact or emotion further increased all ratings.

**Conclusion:**

These findings indicate that disclosing emotions may improve interpersonal interactions, with potential management applications in business.

## Introduction

Good relationships in the workplace have numerous positive effects, including heightened intrinsic motivation (Ryan and Deci, 2000, 2017), enhanced growth and learning (Kram and Isabella, 1985), and a compassionate organizational culture (Sias, 2008; Kark, 2011). They also result in greater employee retention (De Clercq et al., 2020), perhaps by increasing commitment and motivation to stay (Basford and Offermann, 2012), by enhancing job and life satisfaction (Simon et al., 2010; Fay and Kline, 2011), and by boosting the sense of fit between the employee and the organization (Kim et al., 2019). Professionals with good relationships are also likely to perform at higher levels than those who have poor work relationships, since they are more likely to seek help and co-worker support (Friedman et al., 2018; De Clercq et al., 2020), to be creative (Zaitouni and Ouakouak, 2018), to take charge and innovate (Love and Dustin, 2014), and to surpass expectations (Kim et al., 2019). They are also better able to resolve conflicts (Simons and Peterson, 2000) and feel safe to voice opinions and ideas (Stephens et al., 2013). Understanding how to build positive relationships with others in the workplace is thus an important research priority (Sias and Cahill, 1998).

One important mechanism for enhancing closeness in the workplace is self-disclosure (Barasch, 2020; Rimé et al., 2020). Self-disclosure can involve sharing personal details including factual statements (e.g., revealing one’s favorite band) and emotion-related statements (e.g., joy experienced when first attending a concert of that band; Collins and Miller, 1994). When discussing self-disclosure, it is important to emphasize that it involves at least two people: the person sharing and the recipient of the disclosure (Collins and Miller, 1994). For decades, much research has been devoted to studying the impact of self-disclosure on relationships and perceived intimacy, often–but not always–showing that self-disclosure can increase liking and closeness for both the person sharing and the recipient (Jourard, 1959; Cozby, 1973; Wheeless, 1976). Interpersonal exercises that involve systematic sharing of personal information by asking and answering a series of intimate questions with another person also increase closeness (Aron et al., 1997). Moreover, sharing biographical information with another person increases activation in brain regions associated with social processing and may lead to cross-brain synchrony (Cañigueral et al., 2021).

Sharing emotional experiences in particular may be important for building closeness. People spontaneously seek out others to disclose both negative and positive experiences (Rimé et al., 1992; Pennebaker et al., 2001). Doing so can serve multiple functions including strengthening social ties and eliciting support (Rimé et al., 1998; Rimé, 2009).

Notwithstanding potential benefits (Stephens et al., 2013), there has long been a bias against disclosing emotions in the workplace. This reluctance may reflect traditional perceptions of emotions as weakness, and a deeply ingrained distinction between a more “rational” work sphere and a private emotional sphere (Kark, 2011). This, of course, is a distinction that has increasingly been blurred during the COVID-19 pandemic, which required individual’s private and professional lives to interact in unprecedented ways (Galanti et al., 2021). Alongside this development, the business world has seen increasing appeals to greater vulnerability and authenticity in the workplace, particularly for leaders (Oc et al., 2019; Couris, 2020; Ernst & Young LLP, 2021).

Despite these trends, research has yet to disentangle the subtleties of different types of vulnerability in the workplace. Specifically, in previous research, the act of sharing *autobiographical/personal facts* about oneself and the specific act of sharing *emotions* were often studied together, making it difficult to determine the impact of sharing emotions *per se* (Pennebaker et al., 2001). Moreover, not enough is known yet about how various factors may influence the effects of disclosing emotions, including recipients’ attitudes toward emotional expression, the sharer’s gender, and whether one considers the disclosure to be similar to one’s own life experiences. Although closeness is a desirable outcome in a professional setting because it can improve work relationships, people may also value how sharing emotions influences perceptions of warmth, competence, and leadership ability (Cuddy et al., 2011). If sharing emotions increases the degree to which others feel close, but decreases perceptions of competence or leadership ability, people may forgo the benefits of emotional sharing in favor of being perceived as good at their job.

Gender may complicate the interactions between sharing emotions and workplace relationships (Lewis, 2000; Hess et al., 2005; Hess, 2014). Social norms linked to traditional masculinity might lead men to refrain from sharing their emotions in fear of being seen as weak (Möller-Leimkühler, 2002). Ironically, some evidence suggests that when men do share emotions, this is well-received, at least by women in a dating context (Collins and Read, 1990; Collins and Miller, 1994). Women, however, face their own dilemma arising from the stereotype of being overly emotional (Cuddy et al., 2004; Bobbitt-Zeher, 2011; Budig et al., 2016). Thus, it is conceivable that women are particularly at risk of harming their professional standing when disclosing emotions in the workplace.

### Situating our study in the field and theoretical framework

**Figure 1:**
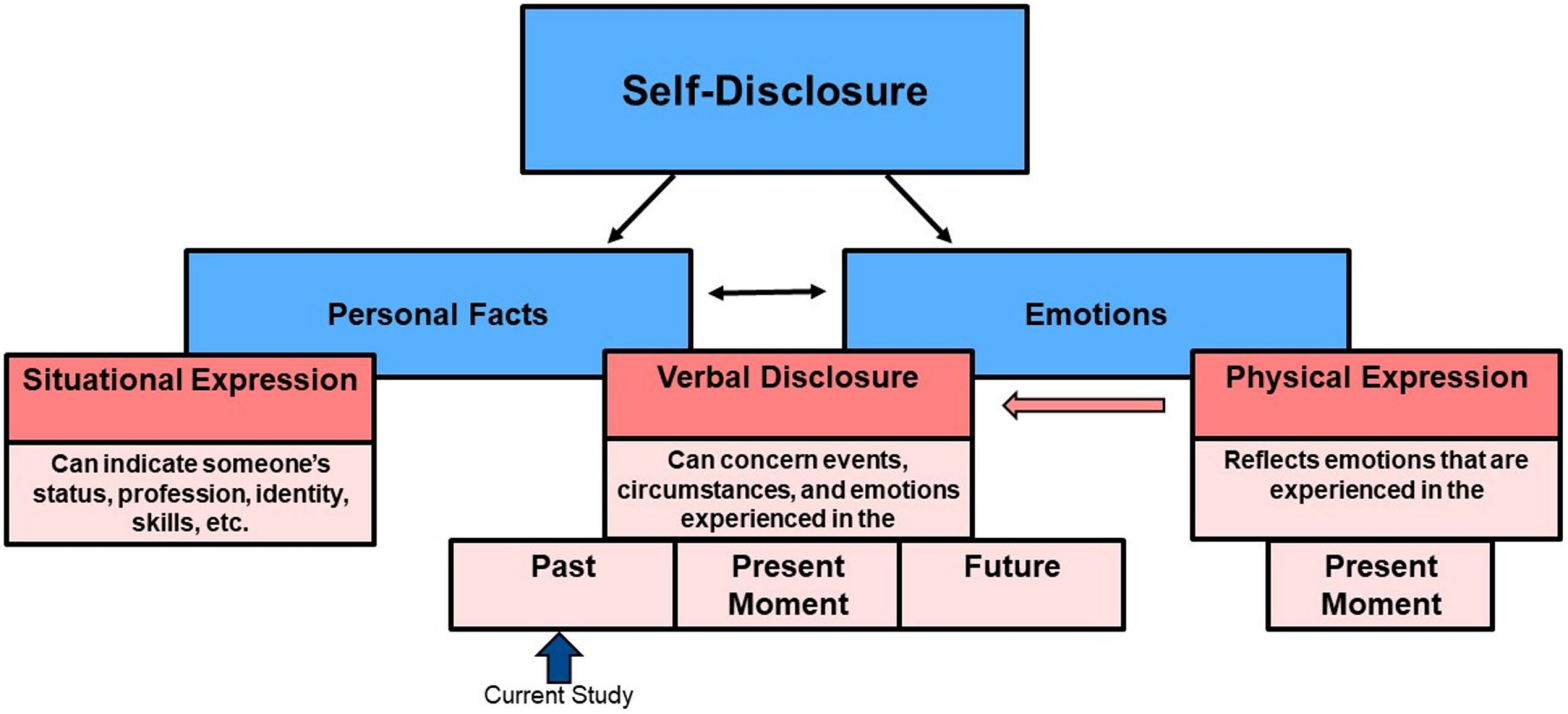
Theoretical Framework. Self-disclosure may involve disclosing emotions and/or personal facts about oneself. In the case of emotions, self-disclosure may occur verbally (e.g., “I feel angry”) or through bodily expressions (e.g., shouting, subtle facial expressions). Personal facts (e.g., autobiographical information) are typically communicated verbally or can be situationally expressed (e.g., through clothes or behavior). One can disclose emotions and personal facts with regards to the past (“When we won the basketball game, I felt very happy”), the present (“When I look at my to-do list, I feel annoyed”), or the future (“I anticipate feeling wonderful when I’ll get married”). The processes of disclosing personal facts and disclosing emotions are often intermingled. For example, certain personal facts may appear to imply specific emotions (e.g., disclosing a loved one’s death typically implies sadness). Nevertheless, reporting a personal fact or event is distinct from explicitly stating how that fact or event makes a person feel. Here, we specifically study the effects of explicit, verbal (written) disclosure of emotions alongside personal facts, when compared to disclosing personal facts alone. The current study assesses self-disclosure of emotions and facts relating to the past rather than the present or the future.

When investigating the impact of emotion sharing, it is important to differentiate between emotional/physical expression, meaning the display of emotions through body language, facial expressions, or tone of voice (Lewis, 2000; Butler et al., 2003; van Kleef, 2014), versus disclosing emotions verbally and factually (see framework in Figure 1 and Lee and Wagner, 2002). Emotional expression typically occurs involuntarily and unconsciously, while verbal disclosure is often a deliberate act. That is, humans can become aware of their emotions and choose to verbally communicate them to a recipient (e.g., “I am sad.”), even if the emotion is not obviously perceptible in concurrent nonverbal cues like facial expressions (Fussell, 2014).

A second consideration is that emotional expression, such as crying, concerns emotions that are *currently* being experienced (if the expression is genuine). Even if the emotion concerns a past event or involves reliving past emotions, the emotion still unfolds in the “here and now.” Verbal disclosure, by contrast, can also refer to emotions experienced in the past or the anticipated future (“Yesterday I felt bad, but now I feel better;” “I anticipate feeling joyful on my wedding day”).

Verbal disclosure of emotions allows people to communicate their inner states calmly, which has advantages—such as peacefully solving interpersonal issues through dialog (e.g., saying “I felt angry about…,” rather than attacking someone physically). Verbal expression of emotions has gained further importance in today’s world because much of communication occurs in written format without witnessing concomitant physical expressions, such as when texting, writing emails, or communicating on social media (Derks et al., 2008; Rimé et al., 2020). For these reasons, we specifically focused on the verbal, textual disclosure of emotions in our study.

The verbal expressions of emotions we used in our study concerned *past* events or circumstances rather than present emotions. Verbal expressions of present emotions are interesting in their own right, but they present additional complexity. Knowing that the sharer is experiencing an emotion *right now* may provoke behavioral responses such as the desire to help or soothe (Christophe and Rimé, 1997). In the case of disclosing emotions about past events, the experience of the emotion itself has already occurred, although it may be re-lived during the act of sharing (Pennebaker et al., 2001). In the current study, we therefore focused on verbally disclosing emotions about *past* events and circumstances.

### Aims and hypotheses

Here we studied the impact of verbally sharing emotions about past events or situations, above and beyond the sharing of personal facts alone, on the recipient’s perceptions of closeness to the sharer. We also investigated potential side effects of sharing emotions on three other factors that are crucial in the interpersonal and professional context, namely perceptions of the sharer’s warmth, competence, and leadership ability, as others have done previously (Zickfeld and Schubert, 2018; Kim and Read, 2021). Additionally, we considered the recipient’s attitudes toward emotions and their personality, as well as the sharer’s gender, and the valence of the emotion disclosed (positive vs. negative).

Specifically, we studied the impact of an individual sharing an emotional experience in addition to an autobiographical fact (e.g., “I often felt sad because my parents fought a lot when I was a child”) compared to sharing *only* the autobiographical fact without emotion (“My parents fought a lot when I was a child”). Importantly, the emotion stimulated by the same event, and thus shared with others, can differ greatly between people and may be positive or negative (e.g., anxious, grateful). Our design allowed us to isolate the effects of positive and negative emotion sharing.

The specific type of self-disclosure studied here involved presentation of a photo of unfamiliar people alongside information about them displayed in written format. This procedure mimicked a common real-life context in which we might read about others online (e.g., a resume, in profiles or blog posts on LinkedIn or other social media platforms). We pre-registered several hypotheses, which are shown in Table 1. Our key hypothesis was that disclosing emotions will foster closeness more than disclosing personal facts alone. In addition, we aimed to assess potential side effects of disclosing emotions on variables crucial to workplace interactions, namely perceived warmth, competence, and leadership ability.

**Table 1:** Pre-registered hypotheses.

Predictions	Reasoning
1. Disclosing emotions about past personal events/circumstances will foster closeness more than disclosing the personal facts alone.^a^	Talking about emotional events increases liking and strengthens social bonds (Pennebaker et al., 2001; Rimé et al., 2020). Emotions are particularly personal experiences to disclose, because they are subjective states.
2. This effect will be diminished or reversed when the recipient holds negative attitudes toward emotional expression.^a^	A recipient with negative attitudes toward emotional expression may harbor judgmental feelings toward anyone who discloses emotions (“emotions as weakness”; see Joseph et al., 1994). This may worsen their opinion of the sharer.
3. Disclosing emotions will increase perceived warmth for both women and men. It will decrease perceived competence in women while perceived competence will be unaffected in men. It will increase perceived leadership ability for men, but reduce it for women.^b^	Various gender differences concerning the effects of emotion disclosure have been documented (e.g., Hess et al., 2005; Brescoll and Uhlmann, 2008; Fischer et al., 2013; Hess, 2014). Women’s emotional expressions appear to sometimes lead to worse professional standing (e.g., Brescoll and Uhlmann, 2008).
4. Effects on closeness, warmth, competence, and leadership ability will differ between types of emotions (i.e., positive vs. negative valence). *Competing hypotheses* (Chamberlin, 1965): Disclosing positive (> negative) emotions will lead to increased ratings for all variables. Disclosing negative (> positive) emotions will lead to increased ratings for all variables (see Yang, 2014; Yang and Kelly, 2016; Barasch, 2020).	These predictions were based on the following, opposing considerations: Disclosing positive emotions does not typically involve confessing experiences that could be perceived as a weakness and might reflect the sharer’s positive outlook on life or resilience. These factors may lead to more positive effects of disclosing positive (> negative) emotions on all outcome variables. However, disclosing negative emotions may be perceived as risky, due to the stigma often associated with them (Businessolver, 2022). Therefore, recipients may feel particularly close to sharers who have the courage to disclose such emotions (Collins and Read, 1990; Collins and Miller, 1994), and they may also rate them as warmer, more competent, and more capable as leaders.
5. Closeness will be predicted by the extent to which the expressed statements are similar to the rater’s own experiences and emotions.^b^	Having something in common with someone increases interpersonal liking (Tsui and O’reilly, 1989; Carli et al., 1991; Byrne, 1997; Devendorf and Highhouse, 2008).
6. Recipients with high openness, low agreeableness, and low neuroticism will show weaker links between similarity and closeness.^b^	These predictions were based on the following considerations (see John and Srivastava, 1999): Open individuals may be more accepting of others’ experiences, even if different from oneself. This openness may lead to feelings of closeness, even in the absence of similarity. Individuals who are not agreeable may show lower feelings of closeness even if similarity is high, as someone with low agreeableness is overall less likely to connect with others on an emotional level. Individuals with low neuroticism may interpret others’ experiences in a more positive way and be less likely to feel stress or anxiety from hearing about experiences different to their own. This positive outlook may lead them to feel closer to someone, even in the absence of similarity.

a We also tested these predictions with regards to warmth, competence, and leadership ability (not explicitly pre-registered), to rule out negative effects of emotion disclosure on these variables that are crucial in the professional context.

b For completeness, analyses on gender and similarity were likewise carried out on all four variables: interpersonal closeness, warmth, competence, and leadership ability.

## Materials and methods

### Transparency and openness

Below, we describe our sampling plan, all data exclusions, all manipulations, and all study measures. Data were analyzed and visualized using R, version 3.6.3 (R Core Team, 2020). Key packages used were *ggpubr* 0.4.0 (Kassambara, 2020), *psych* 1.9.12.31 (Revelle, 2019), *colorspace* 1.4.1 (Stauffer et al., 2015), *cowplot 1.0.0* (Wilke, 2019), *compareGroups* 4.5.1 (Subirana et al., 2014) and packages from the *tidyverse 1.3.1* (Wickham et al., 2019). The data and the main analysis script have been made available on OSF.^1^ The study design and hypotheses were pre-registered.^2^ We made small departures from our pre-registered analysis plan (namely substituting simple linear regressions for linear mixed-effects models) as this led to more accurate parameter estimates given the structure of the data.

### Participants

The final sample consisted of 119 valid participants (64 in the negative emotion condition, 55 in the positive emotion condition), all residing in the United States. Half (55%) were undergraduate or graduate students, the others had various professions (42%, e.g., accountant, teacher, sustainability professional, and counselor) or were unemployed (3%). The composition was 95 female, 23 male, 1 non-binary, age *M* = 25.92, and SD = 9.69; identifying as Asian: 51, White: 47, White and Asian: 4, Black or African American: 11, Asian and other: 1, Other or no answer: 5. Eleven participants indicated that they were Spanish, Hispanic, and/or Latino. Two-hundred participants signed up for the study, but only 164 actually took part when the link was sent out. Out of these, 32 were excluded due to failing at least one of the seven attention checks in the survey, and four could not be matched with the respective experimental data due to missing or faulty IDs. Nine further participants were excluded for taking <1 s on average to complete each of the ratings in the experiment itself (Wood et al., 2017). All exclusion criteria were pre-registered. Initially, we had planned for a sample size of 150 based on a power calculation in GPower 3.1. The reason for the lower achieved sample size was that the number of no-shows and invalid data were higher than anticipated. Participants were recruited online via an invitation by the Wharton Behavioral Lab of the Wharton School at the University of Pennsylvania, which has an existing database of research participants who take part in various experiments. Data collection took place at the end of July 2021. Participants received $6 compensation.

### Procedure

**Figure 2:**
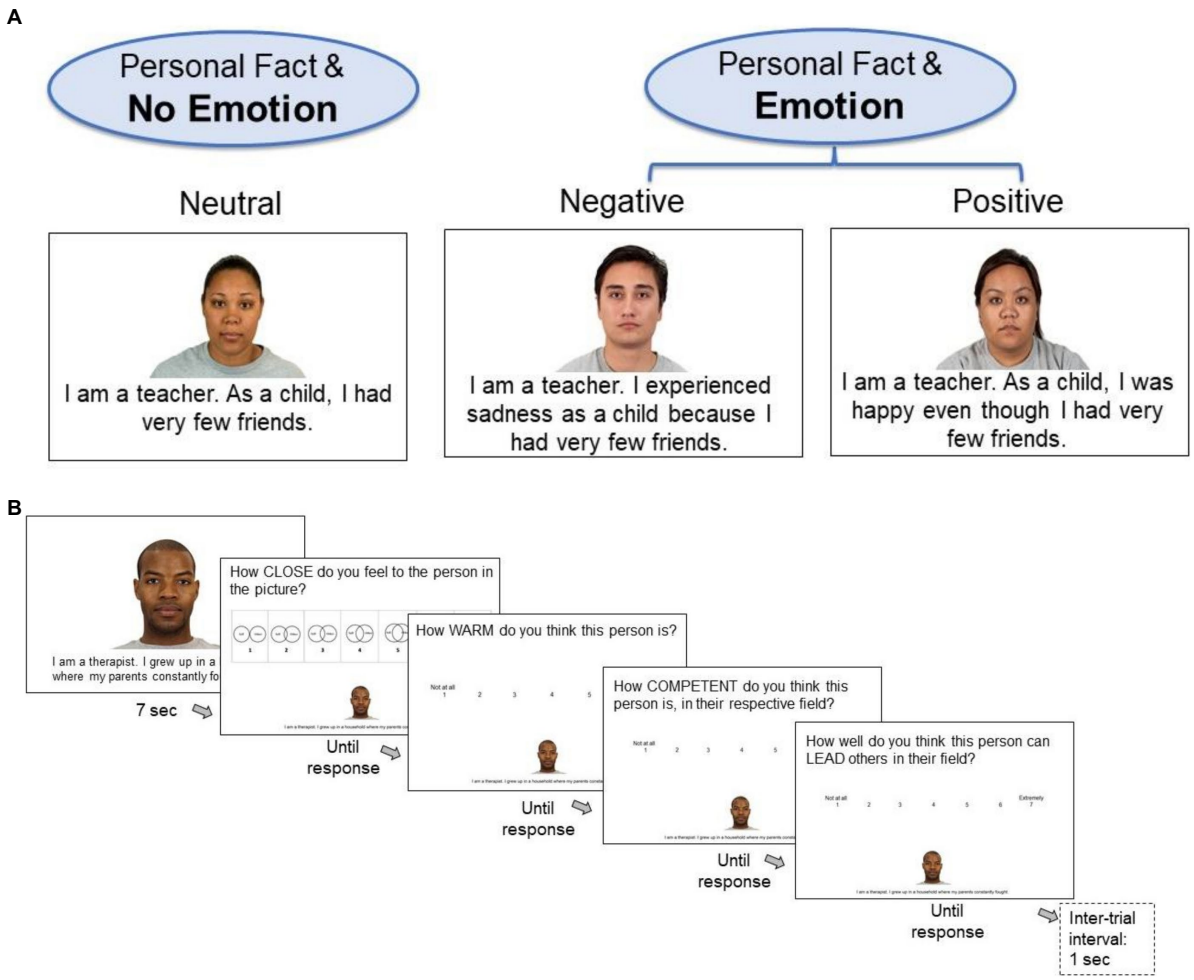
Experimental Design. **(A)** *Example stimuli for the three conditions*. All participants saw personal statements without emotion (NoEmotion condition) in half of the trials of the experiment (16 trials). During the other half of the trials (16 trials), half of participants saw the same statements with a negative emotion and the other half of participants saw them with a positive emotion (Emotion condition). Pictures of faces were kindly provided by the Chicago Face Database (Ma et al., 2015), reproduced with permission. **(B)** *Depiction of one trial*. Participants saw headshots of people with a short statement about their profession, with autobiographical information from their past, and—on Emotion-trials—with the report of a positive or negative emotion with regards to that personal information. They then rated closeness, warmth, competence, and leadership ability of the person.

Participants were randomly assigned to one of two conditions (either positive emotion or negative emotion). They were not made aware that another condition existed. Participants all carried out the study on a laptop/computer at home (not smartphone). First, they completed the pre-experimental surveys in Qualtrics. Second, they clicked on a link which presented the experiment in the Pavlovia research environment (pavlovia.org, see Figure 2). The experiment was created in PsychoPy and the resulting javascript code was further developed outside PsychoPy. Third, after completing the experiment, participants returned to Qualtrics to complete the post-experiment questionnaires. Completing the entire study took ~35 min.

### Stimuli

#### Photos

Participants viewed 32 photos, which showed headshots of different people (*sharers*) from four ethnic groups and two genders (female/male): 50% male; 50% female; 25% for each of four ethnic groups (Asian, Black, Latino, and White). The various ethnic groups were not included to address a particular research question, but rather to ensure diversity of the stimulus material while simultaneously permitting exploratory analyses. Photos were selected from the Chicago Face Database (Ma et al., 2015) for a relatively neutral expression, being 1SD under or above average attractiveness (3.54 ± 0.79 on a scale from 1, not at all, to 7: extremely), and similar age (mean age of person shown in picture: 28.9 years ± 6.3).

#### Statements

Participants also viewed a set of 16 statements accompanying (and ostensibly disclosed by the people portrayed in) the headshots (randomly paired). Statements always included an occupation (e.g., “I am a therapist.”), and autobiographical information (e.g., “I grew up in a household where my parents constantly fought.,” see Table 2). Each statement was shown twice in the experiment, randomly paired with a different face each time. In one of these two instances, it was accompanied by a statement about an emotion. The emotion was always negative for the group assigned to negative emotions, and always positive for the other group (i.e., Valence was a between-subject factor while Emotion vs. NoEmotion was a within-subject factor).

**Table 2:** Text material used in the rating experiment.

Sentence #	Neutral	Negative	Positive
1	I am a surgeon. As a child, my grandfather died from untreatable brain cancer.	I am a surgeon. As a child, I was sad when my grandfather died from untreatable brain cancer.	I am a surgeon. As a child, I was relieved that my grandfather no longer had to suffer when he died from untreatable brain cancer.
2	I am a therapist. I grew up in a household where my parents constantly fought.	I am a therapist. I had anxiety growing up because my parents constantly fought.	I am a therapist. I found my inner strength growing up in a household where my parents constantly fought.
3	I am a researcher. Growing up, my parents pressured me academically.	I am a researcher. Growing up, my parents pressured me academically, resulting in stress.	I am a researcher. I’ve been grateful that my parents pressured me academically growing up.
4	I am a meteorologist. I survived several destructive hurricanes as a child.	I am a meteorologist. I survived several destructive hurricanes growing up, causing anxiety.	I am a meteorologist. I survived several destructive hurricanes growing up, causing me to feel grateful.
5	I am a teacher. As a child, I had very few friends.	I am a teacher. I experienced sadness as a child because I had very few friends.	I am a teacher. As a child, I was happy even though I had very few friends.
6	I am a public speaker. I had a severe stutter when I was growing up, causing difficulties.	I am a public speaker. Growing up, I had a severe stutter that led to difficulties and caused me insecurity.	I am a public speaker. Growing up, I had a severe stutter that taught me to feel confident despite my difficulties.
7	I am a social media influencer. I used to work as a journalist.	I am a social media influencer. I was disappointed in my previous job as a journalist.	I am a social media influencer. I had fun during my previous job as a journalist.
8	I am a vet. When I was a child, we had a dog.	I am a vet. When I was a child, I was afraid of our dog.	I am a vet. When I was a child, playing with our dog made me happy.
9	I am a climatologist. In my job, I’ve been studying climate change.	I am a climatologist. My job studying climate change has sometimes made me fearful.	I am a climatologist. My job studying climate change has sometimes made me hopeful.
10	I am an engineer. I grew up reading books about complex machines.	I am an engineer. Growing up, I was afraid of complex machines.	I am an engineer. Growing up, I was fascinated by complex machines.
11	I am a librarian. I took a gap year before attending college.	I am a librarian. I’ve regretted taking a gap year before attending college.	I am a librarian. I’ve been glad that I took a gap year before attending college.
12	I am a lawyer. I have worked on human rights issues in the past.	I am a lawyer. I have worked on human rights issues in the past, which sometimes made me angry.	I am a lawyer. I have worked on human rights issues in the past, which sometimes made me optimistic.
13	I am postdoctoral researcher. I was working toward my PhD thesis a few years ago.	I am a postdoctoral researcher. I got anxiety from working toward my PhD thesis a few years ago.	I am a postdoctoral researcher. I derived joy from working toward my PhD thesis a few years ago.
14	I am a cook. My parents mainly fed me fast food when I was young.	I am a cook. When I was young, I felt frustrated that my parents mainly fed me fast food.	I am a cook. When I was young, I was amused that my parents mainly fed me fast food.
15	I am a personal trainer. I did not make the basketball team in high school.	I am a personal trainer. I was disappointed when I did not make the basketball team in high school.	I am a personal trainer. I felt determined when I did not make the basketball team in high school.
16	I am a salesperson. I did not attend college.	I am a salesperson. I’ve felt insecure many times because I did not attend college.	I am a salesperson. I’ve been happy that I did not attend college.

Statements were developed by authors BB, JC, and NC in an iterative procedure. Emotion sentences and NoEmotion sentences were matched to be relatively similar in length and amount of information.

### Pre-experiment surveys

Before the experiment began, participants completed the Attitudes toward Emotional Expression scale (AEE) containing 20 items (one reverse-scored; Laghai and Joseph, 2000) and the Big Five Inventory (BFI) containing 44 items (16 reverse-scored; John and Srivastava, 1999). Higher AEE scores denote a more negative orientation toward emotional expression. The BFI measures five dimensions of personality: extraversion vs. introversion, agreeableness vs. antagonism, conscientiousness vs. lack of direction, neuroticism vs. emotional stability, openness vs. closedness to experience. We only made predictions about agreeableness, neuroticism and openness and therefore only used those scores in the analysis.

### Rating experiment

Participants were instructed that we were interested in how people form first impressions, based on similar instructions by Cuddy et al. (2004). We explained that they would see a series of 32 photos of human faces along with brief statements from each person. Participants were asked to form a first impression of each person and then evaluate four attributes. The four attributes were described as follows: “1. Closeness (How close do you feel to the person?), 2. Warmth (comprising traits like kindness, friendliness, trustworthiness, morality, sincerity, etc.,), 3. Competence (comprising efficacy, skill, creativity, confidence, intelligence, etc.,). 4. Leadership (ability to lead others in their field).” The descriptions of warmth and competence were adapted from Cuddy et al. (2008).

We also mentioned that, while it is difficult to make such detailed evaluations based on little information, participants should just report their first impressions without thinking too much. Moreover, to motivate participants to pay attention, they were told that we would ask them a few simple questions afterwards on what they remember about the experiments. The respective picture and sentences were shown for 7 s in full-screen (Figure 2B). Afterwards both the picture and sentences were shown in small format at the bottom part of the screen so that participants could refer back to them while giving their ratings. Responses on the closeness question were given on the Inclusion of Other in the Self Scale (IOS; Aron et al., 1992). This pictorial scale consists of seven response options, each showing two circles labeled as ‘Self’ and ‘Other’, which vary in terms of overlap, from barely touching (1) to almost completely overlapping (7). In the instructions, we acknowledged that it is hard to feel close to a stranger in a picture, but that one may still experience some feelings of closeness. Responses for warmth, competence and leadership were given on 1–7 Likert scales from 1: not at all to 7: extremely. All responses were given via keyboard and participants had an unlimited time to respond.

### Post-experiment surveys

#### Ratings regarding the similarity of all disclosures to one’s own experience and emotions

After the experiment, participants were shown each of the 32 statements from the experiment again, including the Emotion and NoEmotion version of each statement, but without the professional component (“I am a postdoctoral researcher”). The list was shown again in Qualtrics. Participants were asked to indicate for each statement “[…], how similar the statement is to your own life experiences. In other words, to what extent do you feel like something similar or the same has happened to you and/or you felt in a similar way about the event?” If only parts of the statement applied to participants (e.g., they experienced the same event, but they felt differently about it), they were asked to simply choose an intermediate rating. Responses were given on a scale from 0 “(not at all similar/I have never experienced anything like this)” to 6 “(extremely similar/I basically experienced the same).”

#### Demographics

Additional variables included gender, age, self-identified race/ethnicity, country, state, profession.

### Attention checks throughout the study

There were seven attention checks in total distributed throughout the study. That is, we included three multiple-choice questions after the Rating Experiment and before the similarity ratings. These concerned remembering the content of the statements participants just saw. In addition, we also explicitly asked participants whether they had paid attention during the experiment and answered honestly. Finally, we hid three additional attention checks within the surveys (e.g., “Please select option 2”).

### Analysis

We used mixed-effects models to analyze the data, predicting trial-level ratings (closeness, warmth, etc.,) from trial-level condition variables (e.g., Emotion vs. NoEmotion, gender of person sharing the emotion, etc.,) and participant-level variables (e.g., positive v. negative emotion, personality, etc.,), treating trials, stimulus sentences, and stimulus images as nested within participants (i.e., as random effects). The benefits of this approach are that the data are retained in their original trial-by-trial format while the non-independence of data points per participant and specific sentence and image are taken into account, producing more accurate standard error estimates (Judd et al., 2012).

We carried out the analysis using *lmerTest* 3.1.3 (Kuznetsova et al., 2017) with the function *lmer()*, and created output tables and plots using *sjPlot 2.8.1* (Lüdecke, 2018) in R. While various models are conceivable, we formulated our models based on our hypotheses. That is, we only included variables and interaction terms that were relevant to the hypotheses. For example, we had predicted an interaction between three personality traits and similarity ratings per sentence but no interactions between personality and condition, emotion type or other variables. Therefore, the latter interaction terms were not included. This was done to avoid overly-complex, hard-to-interpret and overfitted models.^3^ Our data are available online should readers wish to analyze the data further. Whenever we included interaction effects between variables, the corresponding main effects and lower-level interaction terms were also included to keep interaction effects interpretable. We also included random intercepts for participant, sentence, and stimulus image, but no random slopes as this led to convergence issues. Thus the model was formulated as follows:


$$\begin{array}{l} \text{Outcome \textasciitilde{} Condition}+\text{Valence}+\text{Attitudes\_Emotions}+\\ \text{Sharer\_Gender}+\text{Similarity}+\text{Openness}+\text{Agreeableness}+\\ \text{Neuroticism}+\text{Condition}*\text{Valence}+\text{Condition}*\\ \text{Attitudes\_Emotions}+\text{Condition}*\text{Sharer\_Gender}+\\ \text{Similarity}*\text{Openness}+\text{Similarity}*\text{Agreeableness}+\\ \text{Similarity}*\text{Neuroticism}+(1|\text{Image})+\\ \left(1|\text{Sentence})+(1|\text{Participant}\right) \end{array}$$

All variables were Z-scored before modeling. Outcome refers to Closeness, Warmth, Competence or Leadership Ability, modeled separately.

**Figure 3:**
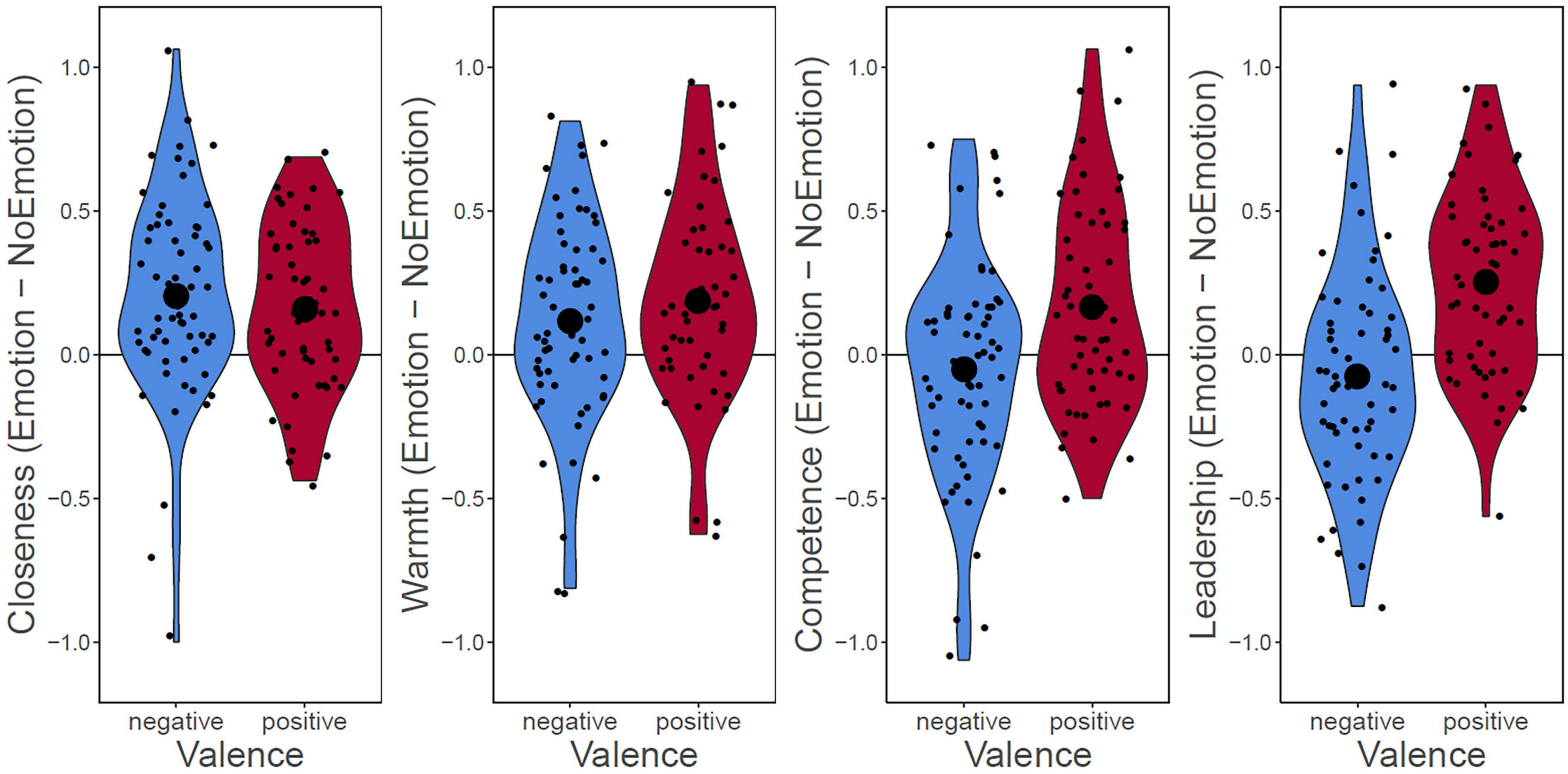
Violin Plots of Effects of Sharing Emotions on the Four Outcome Variables for (Red) Positive and (Blue) Negative Emotions. To create this figure, the difference between the Emotion and NoEmotion version of each sentence was calculated for each outcome variable. Then, an average difference score per participant and outcome variable was calculated across the 16 sentences. Each dot represents the result for one participant. The large dot represents the mean across participants. Figure serves visualization only, as the analysis was conducted using mixed-effects models on the trial-by-trial data. *n* = 119.

For visualization purposes only, we also calculated the difference in raw ratings between the Emotion and NoEmotion versions of each sentence per participant (Difference = Emotion − NoEmotion), and subsequently averaged these values across the 16 sentences per participant. The resulting mean difference scores per outcome variable and valence are shown in Figure 3.

## Results

### Descriptive values

Participants assigned to the two groups (positive vs. negative) did not differ significantly in terms of age, gender, racial self-identification, or AEE scores (see Table 3). Means, SDs, ranges, and Cronbach’s alpha for the questionnaire measures employed in this study (AEE and BFI) are shown in Table 4. Figure 3 depicts average differences in raw ratings between statements containing Emotion vs. NoEmotion per type of valence.

**Table 3:** Summary descriptives by experimental group.

	Negative emotion condition	Positive emotion condition	*p* overall
	*N* = 64	*N* = 55	
Age	25.3 (9.07)	26.6 (10.4)	0.476
Gender			0.107
Female	47 (73.4%)	48 (87.3%)	
Male	16 (25.0%)	7 (12.7%)	
Non-binary	1 (1.56%)	0 (0.00%)	
Race			0.326
Asian	29 (46.0%)	22 (41.5%)	
Black or African American	3 (4.76%)	8 (15.1%)	
White	27 (42.9%)	20 (37.7%)	
Asian and Other	0 (0.00%)	1 (1.89%)	
White and Asian	3 (4.76%)	1 (1.89%)	
Other	1 (1.59%)	1 (1.89%)	
Attitudes toward Emotional Expression scale (AEE)	47.8 (11.5)	49.6 (13.0)	0.411

Three participants did not select a race and are not included here. *p*-values refer to the comparison between groups using chi-square tests or independent *t*-tests, as appropriate.

**Table 4:** Descriptive values for the questionnaire tools employed.

	Mean	SD	Observed range		Possible range		Cronbach’s
			Min	Max	Min	Max	Alpha
Attitudes toward Emotional Expression (AEE) scale	48.61	12.18	23.00	79.00	20	100	0.89
BFI-Agreeableness	3.66	0.67	2.00	4.89	1	5	0.83
BFI-Neuroticism	3.06	0.86	1.00	5.00	1	5	0.87
BFI-Openness	3.54	0.68	1.70	4.90	1	5	0.84
BFI-Extraversion	3.02	0.84	1.00	5.00	1	5	0.87
BFI-Conscientiousness	3.81	0.63	1.56	5.00	1	5	0.84

BFI, Big Five Inventory. *n* = 119.

### Results of multilevel modeling approach

**Figure 4:**
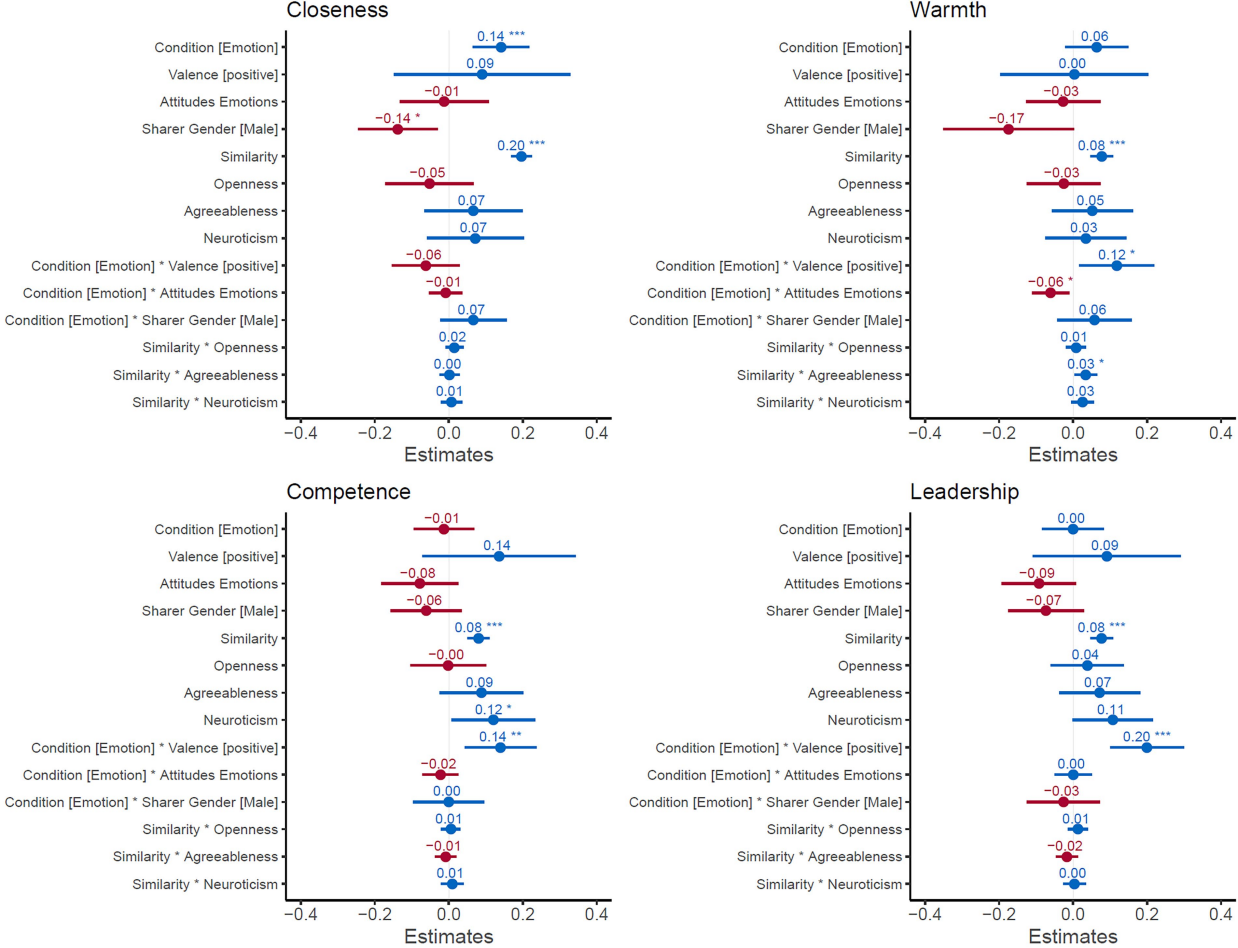
Mixed-Effects Models Predicting the Four Outcome Variables Based on Variables of Interest. Shown are the beta weights with 95% confidence intervals. Condition (Emotion vs. NoEmotion), valence, sharer gender, and similarity ratings are trial-level variables. Attitudes, Openness, Agreeableness, and Neuroticism are participant-level variables. Random effects included random intercepts for participant, stimulus picture, and sentence. Plot was created using the *sjPlot* package in R.

Figure 4 depicts the results for the mixed-effects models including the beta estimates (fixed effects only). All values for the models can also be found in Supplementary Table 1. The models (including both fixed and random effects) explained 49%, 38%, 43%, and 40% of the variance in the data for closeness, warmth, competence, and leadership, respectively (conditional *R*^2^-values).

#### Disclosing emotions increased ratings of closeness

There was a main effect of Condition (Emotion > NoEmotion) on perceived closeness, but no interaction of Condition with Valence (Positive vs. Negative). This indicates that positive and negative sharing increased closeness to a similar extent.

#### Disclosing positive emotions increased ratings of warmth, competence, and leadership ability

There were no significant main effects of disclosing emotions on warmth, competence, and leadership in the full model. However, there were significant interaction effects of Condition with Valence: Disclosing positive emotions led to significantly increased ratings of warmth, competence and leadership ability, relative to negative emotions (Figure 3).

#### Attitudes toward emotional expressions were largely unrelated to outcomes

**Figure 5:**
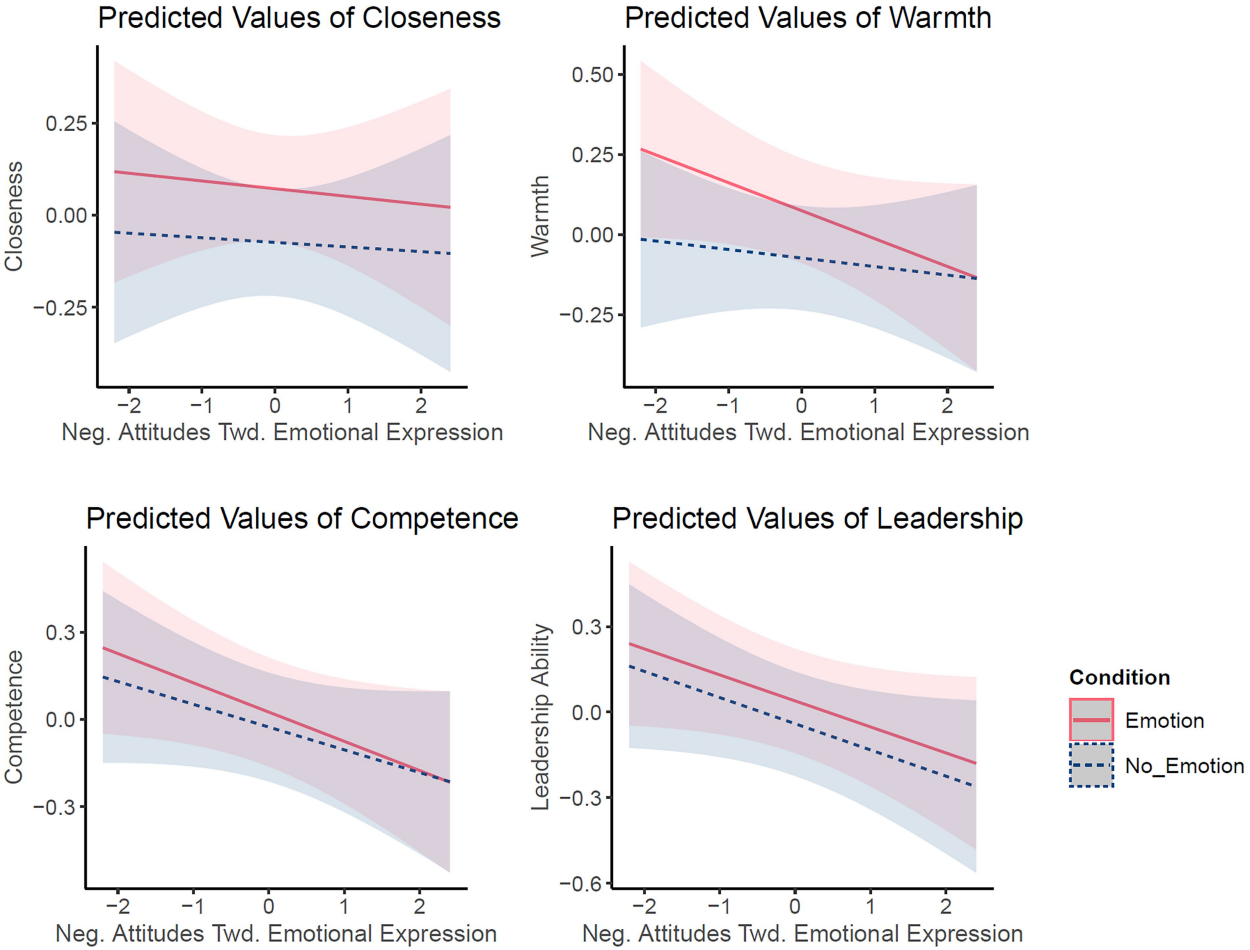
Values of All Outcome Variables as Predicted by Negative Attitudes Toward Emotions and Condition (Emotion vs. NoEmotion). Plot was created using the *sjPlot* package in R based on the reported model. Lines shown depict the predicted values, not data, to visualize the model. The other discrete predictors, not plotted here, were held constant at their proportions (not reference level). All values are Z-scored.

There was only one significant interaction effect between Attitudes toward Emotional Expression and Condition for Warmth ratings: Participants with more negative attitudes toward emotional expression gave lower warmth ratings when an emotion (vs. no emotion) was disclosed, but the effect size was small (standardized beta = −0.06, *p* = 0.02, and see Figure 5). For all other effects on closeness, competence, and leadership ability, there were no interaction effects of Condition (Emotion vs. NoEmotion) with attitude scores, indicating that attitudes did not influence effects.

#### The effects of sharing emotions did not vary with gender

Contrary to our predictions, it did not matter whether a man or a woman disclosed emotions: there were no significant interaction effects of sharer’s gender or condition (Emotion > NoEmotion) for any of the outcome variables. However, there was a main effect of sharer’s gender for closeness in that participants reported feeling closer to women than men.

#### Having something in common with a sharer influenced all ratings

Finally, the degree to which participants had similar experiences in common with the person shown in the headshot significantly predicted all of the variables. This effect was particularly pronounced for closeness ratings (standardized beta = 0.20, *p* < 0.001).

More agreeable participants showed slightly stronger effects of similarity on perceived warmth, but the effect size was very small (standardized beta = 0.03, *p* = 0.03). Otherwise, openness, agreeableness and neuroticism did not interact with similarity ratings.

## Discussion

### Summary and implications

Emotions and vulnerability are increasingly important topics in management and business (Ashkanasy and Dorris, 2017; Navas and Vijayakumar, 2018; Kock et al., 2019). This trend has been accelerated by the COVID-19 pandemic (Nielsen et al., 2020), in part by diluting boundaries between professional and personal lives when working from home, and by growing recognition that employees’ mental health is critical for attracting and retaining talent (Meissner et al., 2020; Sull et al., 2022). Employees who felt more connected with their co-workers during the pandemic were also two to three times more productive on collaborative tasks (Dahik et al., 2020). Our study, conducted in the midst of the pandemic, shows that explicitly sharing emotions (both positive and negative) about past events can have beneficial effects on interpersonal closeness. Sharing positive emotions specifically also increased perceived warmth, competence and leadership ability. Sharing negative emotions did not have any significant positive or negative effects on these factors (except for a positive trend for warmth). Our findings indicate that it may be both safe and beneficial to verbally disclose emotions about past events in a workplace context. Our study adds to the existing literature by specifically investigating the interpersonal impact of explicit, textual sharing of emotions above and beyond the sharing of personal facts or experiences alone, two factors that have often been intermingled in previous research (Barasch, 2020).

Notably, the impacts of sharing emotions–in addition to personal facts--did not depend on the gender of the sharer. This was contrary to our predictions and to commonly held stereotypes about women and men in the workplace, as well as some previous research (Brescoll and Uhlmann, 2008; Fischer et al., 2013). For example, Brescoll and Uhlmann (2008) showed that if women express anger in the workplace they are perceived as having lower status, while the same is not true for men (Hess et al., 2005; Hess, 2014). Our findings offer promise that both women and men may reap the benefits of vulnerability in the workplace. However, it should be noted that a large proportion of participants (77%, i.e., the recipients of the disclosures) in the current study identified as female. Future research should collect more data from male participants.

We also found that the impact of recipients’ own attitudes toward emotional expression were negligible (Laghai and Joseph, 2000). Importantly, even if the recipient of the disclosure had excessively negative attitudes toward emotional expression, emotional sharing was not harmful. We only found a very small effect for warmth, in that people with more negative attitudes toward emotional expression rated individuals as slightly less warm when they shared emotions (vs. no emotions). Thus, any negative effects were negligible and outweighed by the beneficial effects on participants with more positive attitudes.

Finally, we found that having something in common with an individual’s disclosure significantly predicted closeness, warmth, competence, and leadership ability, which is in accord with prior findings (Carli et al., 1991; Devendorf and Highhouse, 2008). The effect was particularly pronounced for closeness. In contrast to what we predicted, personality did not modify this relationship, except for a very small effect for agreeableness: Agreeable participants showed slightly stronger effects of perceived similarity on warmth (not corrected for multiple comparisons), which is in line with our predictions. While the finding of similarity predicting closeness is not surprising, the finding of similarity predicting ratings of warmth, competence and leadership ability are more consequential. A bias for perceiving individuals with similar experiences as more professional may sometimes lead to the upholding of racist and sexist biases in the workplace, insofar these experiences are widely shared by individuals of a particular gender or from a specific ethnic group. Despite this potential danger, this finding invites the possibility of improving connections between ethnic and gender groups by disclosing personal and emotional experiences that are common to everyone.

The beneficial effects of expressing positive emotions on perceived competence and leadership are noteworthy. Harker and Keltner (2001) likewise found that the degree of positive emotional expression in women’s college yearbook pictures was correlated with observer ratings of these women on several traits including competence. One possibility is that expressing positive emotions (e.g., gratitude) about personal experiences signals that one has the ability to learn and grow from hardship, and to discover the silver lining in difficult situations, which may signal competence and leadership potential (Spreitzer, 2006; Gonzalez, 2010). Some of our positive sentences were indeed explicitly worded in a way that suggested resilience (e.g., ‘I was happy even though I had very few friends.’). Fredrickson (2004) proposed the broaden-and-build theory of positive emotions, whereby positive emotions are theorized to help build resources and skills for the future. Wong et al. (2013) found that expressing positive emotions promotes goal-attainment in workplace interactions, but only when expressed to superiors rather than colleagues. Moreover, the ways individuals respond to their romantic partners when discussing positive events is more predictive of well-being and breaking up 2 month later than are their responses to negative events (Gable et al., 2006).

Research on “general leader trait affect,” rather than the verbal expression of specific emotions, has also revealed some benefits of positive over negative emotions for leadership. Joseph et al. (2015) showed that positive leader affect predicts several leadership traits, including transformational leadership, and leadership effectiveness, whereas negative leader affect shows the inverse relationship (Gaddis et al., 2004). Leader affect can impact follower affect via emotional contagion and leaders who express positive emotions might also be perceived as charismatic (Johnson, 2008; Rajah et al., 2011).

Our findings can be applied to conflict mediation, team-building exercises, and leadership training. They may also be relevant in other contexts, for example in doctor–patient interactions that depend on trust to work. We note that disclosing emotions requires psychological safety and trust in the work environment (Frazier et al., 2017; Newman et al., 2017). By extension, it seems possible that disclosing emotions in a toxic work environment may be harmful, although it is also conceivable that it may help to *reduce* toxicity by increasing compassion, trust, and connection. Notably, the ability to disclose emotions effectively is a skill that can be learned. A recent study by the Boston Consulting Group indicates that a 10-week mindfulness training fosters the ability to recognize and describe emotions, as well as the ability to non-judgmentally listen to others’ experiences (Meissner et al., 2020).

### Limitations and future research

Our study has a number of limitations. First, as mentioned, our sample was primarily female, young, White and Asian. It is possible that individuals from other demographic groups react differently to emotion disclosure. For example, since men tend to talk about emotions less than women do (Pennebaker et al., 2001; Brody et al., 2016), it is conceivable that they will react to self-disclosure less positively than did our largely female sample. Future studies should explore if our findings extend to other groups.

Second, the set-up might have been perceived as somewhat artificial and some participants might have guessed what the experiment was about. The use of standardized, artificial narratives and images mimicked the now common situation of learning about unfamiliar people by reading online professional networking sites or using resume sharing services like LinkedIn. Due to this design, our study may have limited generalizability to in-person interactions or to relationships with known individuals (e.g., colleagues). Concerning the risk of participants guessing the objectives of the study, it should be noted that there were numerous factors in the experiment (e.g., gender and race of the sharer, complex sentences about the sharer’s past). Therefore, it would have been difficult for participants to determine precisely our motives and interests, which somewhat mitigates concerns about demand characteristics. Participants were also blinded to the existence of the other emotion condition (i.e., participants randomly assigned to the positive emotion condition did not know about the existence of a negative emotion condition). Finally, even if a participant understood the purpose of the study that does not necessarily invalidate the results (e.g., someone commented: “It was interesting how the slight change in words in some of the sentences makes you change your rating.”). To increase ecological validity, future studies should conduct a similar experiment in real-life conditions and should include observable behavior as an outcome variable, in addition to self-reports.

The third limitation concerns the neutral expression of the person in the photographs while speaking about positive or negative emotions. This type of emotional incongruence or suppression is often viewed negatively (Butler et al., 2003). However, this approach permitted us to standardize the experiment, because–unlike many previous studies–we were not interested in the impact of emotional expression *per se* (e.g., breaking out into tears), but in the verbal disclosure of emotions. Moreover, since individuals were speaking about emotions they experienced in the past rather than the present, a neutral facial expression is still realistic.

Fourth, one may argue that the emotion-disclosure condition simply included a higher degree of self-disclosure, which could be driving the effects (rather than emotion *per se*). We attempted to keep the sentences matched in length compared to the NoEmotion condition, but this is still a valid concern. Future studies could address this issue by increasing the length of the NoEmotion condition sentences.

Finally, we note that our study does not concern the experience or expression of emotions *per se*, but rather verbal information about emotions. Our findings are not directly translatable to the physical expression of emotions, because emotional expression (e.g., shouting when angry, crying when sad) might have very different effects on recipients compared to a factual statement about emotions. Our design also cannot distinguish effects of specific emotions (e.g., anger, sadness) but only concerns the valence of the reported emotions (positive vs. negative). It is highly plausible that effects also depend on the specific emotions experienced (Brans et al., 2014). For example, research has shown that, for women, expressing anger results in being perceived as lower status, while this is not true for men (Brescoll and Uhlmann, 2008). These questions should be further addressed in future research. Given the beneficial effects of disclosing positive emotions on all outcomes in our study, it may be particularly interesting to disentangle the effects of disclosing distinct positive sentiments (e.g., gratitude, joy, excitement, curiosity) on the way people are perceived and treated at work.

Future research should aim to uncover precisely when and where the expression of specific emotions is useful or harmful. In the “new normal” of hybrid and remote work, it will be crucial to find ways to connect with one another in meaningful and authentic ways. The current study focused on expressing emotions about past emotional experiences rather than current ones, which may be an important distinction. Either way, the authenticity of the emotional expression likely matters (see Ceri-Booms, 2010; Crawford et al., 2019; Oc et al., 2019).

### Concluding remarks

Our study was carried out in the midst of the global COVID-19 pandemic, which may have changed the importance and role of emotions and vulnerability in the workplace (Daraba et al., 2021). Rates of anxiety, uncertainty, and loneliness were at or near record levels (Czeisler et al., 2020; Usher et al., 2020). Leaders who did not acknowledge and disclose these emotions may have been perceived to be out-of-touch, possibly contributing to the “Great Resignation” (Sull et al., 2022). Moreover, the unprecedented shift to remote work led professionals–even managers–to show themselves in authentic and vulnerable settings when attending online meetings from their homes (Galanti et al., 2021). The shared experience of the COVID-19 pandemic has made it more acceptable for all of us to show our humanity, which includes communicating our emotional experiences. Our study shows that doing so increases interpersonal closeness above and beyond the effects of sharing personal facts alone, and that sharing positive emotions specifically increases perceptions of warmth, competence and leadership ability.

## Data availability statement

The datasets presented in this study can be found in online repositories. The names of the repository/repositories and accession number(s) can be found at: https://osf.io/sp6rm/?view_only=e95ec1dfcc864a458c666a72164faee1.

## Ethics statement

The studies involving human participants were reviewed and approved by the IRB of the University of Pennsylvania. The participants provided their written informed consent to participate in this study.

## Author contributions

VL, BB, JC, NC, and MP designed the study. BB, JC, and NC prepared the sentences used in the experiment. JC selected the stimulus pictures. NC programmed the Pavlovia experiment and randomization, with help from JC. BB and VL selected and prepared the online questionnaires and performed the data collection. VL analyzed the data and created the figures of results, with support from the other authors, particularly DC. VL, BB, JC, NC, DC, and MP carried out literature review. VL, BB, DC, and MP interpreted the data and drafted the manuscript. All authors contributed to the article and approved the submitted version.

## Funding

The research was supported by the Wharton Dean’s Research Fund and the Wharton Behavioral Lab (VL and MP), the R37-MH109728, R01-MH108627, R01-MH-118203, KA2019-105548, U01MH121260, R01-NS123054, R21AG073958, UM1MH130981, R56MH122819, and R56AG071023 (MP), the Summer Undergraduate Internship Program (SUIP) at the University of Pennsylvania (BB), and the Penn Undergraduate Research (PURM) mentoring program (JC and NC).

## Conflict of interest

The authors declare that the research was conducted in the absence of any commercial or financial relationships that could be construed as a potential conflict of interest.

## Publisher’s note

All claims expressed in this article are solely those of the authors and do not necessarily represent those of their affiliated organizations, or those of the publisher, the editors and the reviewers. Any product that may be evaluated in this article, or claim that may be made by its manufacturer, is not guaranteed or endorsed by the publisher.

## Footnotes

^1^ https://osf.io/sp6rm/?view_only=e95ec1dfcc864a458c666a72164faee1

^2^ https://aspredicted.org/k84u9.pdf

^3^We did not include the race and gender of participants and their interactions with the race and gender of the person disclosing the information. While interesting, such analysis would have been underpowered, as there were not enough cases per subcategory (e.g., Pictures of White Female Individuals with an Emotion-statement, or male study participants overall).
