# Supplementary material for: The impact of disclosing emotions on ratings of interpersonal closeness, warmth, competence, and leadership ability
Source: Front Psychol. 2022 Dec 13;13:989826. doi: 10.3389/fpsyg.2022.989826 (PMC9793093; doi:10.3389/fpsyg.2022.989826)
Supplement: Supplementary file 1 [file Data_Sheet_1.PDF]

	IOS_Z_m1			Warmth_Z_m1			Competence_Z_m1			Leadership_Z_m1		
Predictors	*Estimates*	*CI*	*p*	*Estimates*	*CI*	*p*	*Estimates*	*CI*	*p*	*Estimates*	*CI*	*p*
(Intercept)	-0.05	-0.24 – 0.14	0.632	0.01	-0.19 – 0.22	0.906	-0.06	-0.27 – 0.16	0.596	-0.05	-0.26 – 0.16	0.666
Condition [Emotion]	0.14	0.07 – 0.22	**<0.001**	0.06	-0.02 – 0.15	0.140	-0.01	-0.09 – 0.07	0.756	0.00	-0.08 – 0.08	0.993
Valence [positive]	0.09	-0.15 – 0.33	0.457	0.00	-0.20 – 0.20	0.971	0.14	-0.07 – 0.34	0.197	0.09	-0.11 – 0.29	0.369
Attitudes Emotions	-0.01	-0.13 – 0.11	0.838	-0.03	-0.13 – 0.07	0.605	-0.08	-0.18 – 0.03	0.139	-0.09	-0.19 – 0.01	0.073
Sharer Gender [Male]	-0.14	-0.24 – -0.03	**0.012**	-0.17	-0.35 – 0.00	0.053	-0.06	-0.16 – 0.03	0.213	-0.07	-0.18 – 0.03	0.159
Similarity	0.20	0.17 – 0.22	**<0.001**	0.08	0.05 – 0.11	**<0.001**	0.08	0.05 – 0.11	**<0.001**	0.08	0.05 – 0.11	**<0.001**
Openness	-0.05	-0.17 – 0.07	0.392	-0.03	-0.12 – 0.07	0.620	-0.00	-0.10 – 0.10	0.976	0.04	-0.06 – 0.14	0.438
Agreeableness	0.07	-0.07 – 0.20	0.324	0.05	-0.06 – 0.16	0.350	0.09	-0.03 – 0.20	0.127	0.07	-0.04 – 0.18	0.195
Neuroticism	0.07	-0.06 – 0.20	0.283	0.03	-0.07 – 0.14	0.533	0.12	0.01 – 0.23	**0.036**	0.11	-0.00 – 0.22	0.051
Condition [Emotion] * Valence [positive]	-0.06	-0.15 – 0.03	0.178	0.12	0.02 – 0.22	**0.022**	0.14	0.04 – 0.24	**0.005**	0.20	0.10 – 0.30	**<0.001**
Condition [Emotion] * Attitudes Emotions	-0.01	-0.05 – 0.04	0.712	-0.06	-0.11 – -0.01	**0.019**	-0.02	-0.07 – 0.03	0.366	0.00	-0.05 – 0.05	0.980
Condition [Emotion] * Sharer Gender [Male]	0.07	-0.02 – 0.16	0.148	0.06	-0.04 – 0.16	0.255	0.00	-0.10 – 0.10	1.000	-0.03	-0.12 – 0.07	0.613
Similarity * Openness	0.02	-0.01 – 0.04	0.220	0.01	-0.02 – 0.04	0.544	0.01	-0.02 – 0.03	0.663	0.01	-0.01 – 0.04	0.333
Similarity * Agreeableness	0.00	-0.03 – 0.03	0.892	0.03	0.00 – 0.06	**0.027**	-0.01	-0.04 – 0.02	0.571	-0.02	-0.05 – 0.01	0.280
Similarity * Neuroticism	0.01	-0.02 – 0.04	0.607	0.03	-0.01 – 0.06	0.104	0.01	-0.02 – 0.04	0.545	0.00	-0.03 – 0.03	0.800
**Random Effects**												
σ^2	0.50			0.62			0.57			0.60		
τ_{00}	$0.39_{\text{Participant}}$			$0.26_{\text{Participant}}$			$0.28_{\text{Participant}}$			$0.26_{\text{Participant}}$		
	$0.02_{\text{Image\_file}}$			$0.05_{\text{Image\_file}}$			$0.01_{\text{Image\_file}}$			$0.01_{\text{Image\_file}}$		
	$0.02_{\text{Sentence\_no}}$			$0.04_{\text{Sentence\_no}}$			$0.10_{\text{Sentence\_no}}$			$0.09_{\text{Sentence\_no}}$		
ICC	0.46			0.37			0.41			0.38		
N	$32_{\text{Image\_file}}$			$32_{\text{Image\_file}}$			$32_{\text{Image\_file}}$			$32_{\text{Image\_file}}$		
	$16_{\text{Sentence\_no}}$			$16_{\text{Sentence\_no}}$			$16_{\text{Sentence\_no}}$			$16_{\text{Sentence\_no}}$		
	$119_{\text{Participant}}$			$119_{\text{Participant}}$			$119_{\text{Participant}}$			$119_{\text{Participant}}$		
Observations	3808			3808			3808			3808		
Marginal R^2 / Conditional R^2	0.053 / 0.492			0.025 / 0.382			0.038 / 0.429			0.040 / 0.403		
AIC	8667.797			9462.925			9134.823			9303.157		
